# Two Novel Vocalizations Are Used by Veeries (*Catharus fuscescens*) during Agonistic Interactions

**DOI:** 10.1371/journal.pone.0120933

**Published:** 2015-03-23

**Authors:** Kara L. Belinsky, Claire E. Nemes, Kenneth A. Schmidt

**Affiliations:** 1 Biology Department, State University of New York New Paltz, New Paltz, New York, United States of America; 2 Department of Biology, Ball State University, Muncie, Indiana, United States of America; 3 Department of Biological Sciences, Texas Tech University, Lubbock, Texas United States of America; Utrecht University, NETHERLANDS

## Abstract

Avian vocalizations are common examples of the complex signals used by animals to negotiate during agonistic interactions. In this study, we used two playback experiments to identify agonistic signals in a songbird species with several acoustically complex songs and calls, the veery. In the first experiment, we compared veery singing behavior in response to simulated territorial intrusions including playback of three variations of veery song: 1) song alone as a control, 2) songs with added whisper calls, and 3) songs with introductory notes removed. In the second experiment, we used multimodal stimuli including songs, whisper calls and songs with introductory notes removed, along with a robotic veery mount. Focal males readily responded to all of the playback stimuli, approached the speaker and/or robotic mount, and vocalized. Male veeries gave more whisper calls, and sang more songs without the introductory note in response to all types of playback. However, veeries responded similarly to all types of stimuli presented, and they failed to physically attack the robotic mount. These results indicate that rival veeries use two different types of novel vocalizations: whisper calls and songs lacking the introductory note as agonistic signals, but do not allow us to discern the specific functions of these two vocalizations.

## Introduction

Vocalizations are commonly observed examples of agonistic signals that animals use during conflicts over resources [1]. Agonistic signals allow competitors to assess each other’s relative fighting motivation or ability, and to avoid costly physical contests when one competitor chooses to retreat rather than fight [2]. For example, the vocalizations of red deer, *Cervus elaphus* [3], and common toads, *Bufo bufo* [4], signal body size, which is a good indicator of fighting ability in these species. The vocalizations of songbirds are a fruitful model for studying agonistic signaling because these birds have complex vocal repertoires, often including a variety of learned songs and largely innate calls that are often used to signal aggressive intent.

Many studies suggest that songbird species use their repertoires of songs and calls to negotiate with their rivals in a variety of ways [5]. Songbird species may be able to signal aggressive intent by song overlapping [6–8]. Species with song repertoires can match the song type, perform another shared song type (repertoire match), or match the frequency of the preceding song produced by their opponent [9–12]. In addition, the performance of physically demanding trilled songs [13, 14], songs with altered acoustic structure[15, 16] or vocalizations given at low amplitudes [17–23] have been shown to signal aggression in a growing list of species. In addition to producing songs, songbird species also produce varying numbers and types of calls, which may be defined as vocalizations that are shorter in duration and simpler in acoustic structure than songs are, although these vocalizations are also sometimes distinguished based on their development (learned or innate), or function (for mate attraction and territory defense, or more general use). The study of songbird call function has focused primarily on the use of calls as signals warning against predators [24, 25] and contacting or identifying group members [26], although calls, like songs, are frequently performed in agonistic contexts.

In their review of agonistic vocal signaling in songbirds, Searcy and Beecher [5] describe three criteria for identifying aggressive signals. The three criteria are: 1) the context criterion, which identifies signals given by the sender in an aggressive context, 2) the response criterion, which identifies signals that elicit an aggressive response from the receiver, and 3) the predictive criterion, which identifies signals that predict acts of aggression by the sender. Many studies have identified agonistic signals using one or more of the three criteria. In this study, we used a playback experiment to identify vocal signals of agonism in a songbird species with acoustically complex songs and calls, the veery (*Catharus fuscescens*). Our playback experiments were designed to use the context criterion to identify agonistic signals, the response criterion to compare differences in the effects of two potential aggressive signals sung by veeries, and the predictive criterion by offering singing veeries the opportunity to follow up aggressive signaling with physical acts of aggression towards a taxidermic mount.

**Fig 1:**
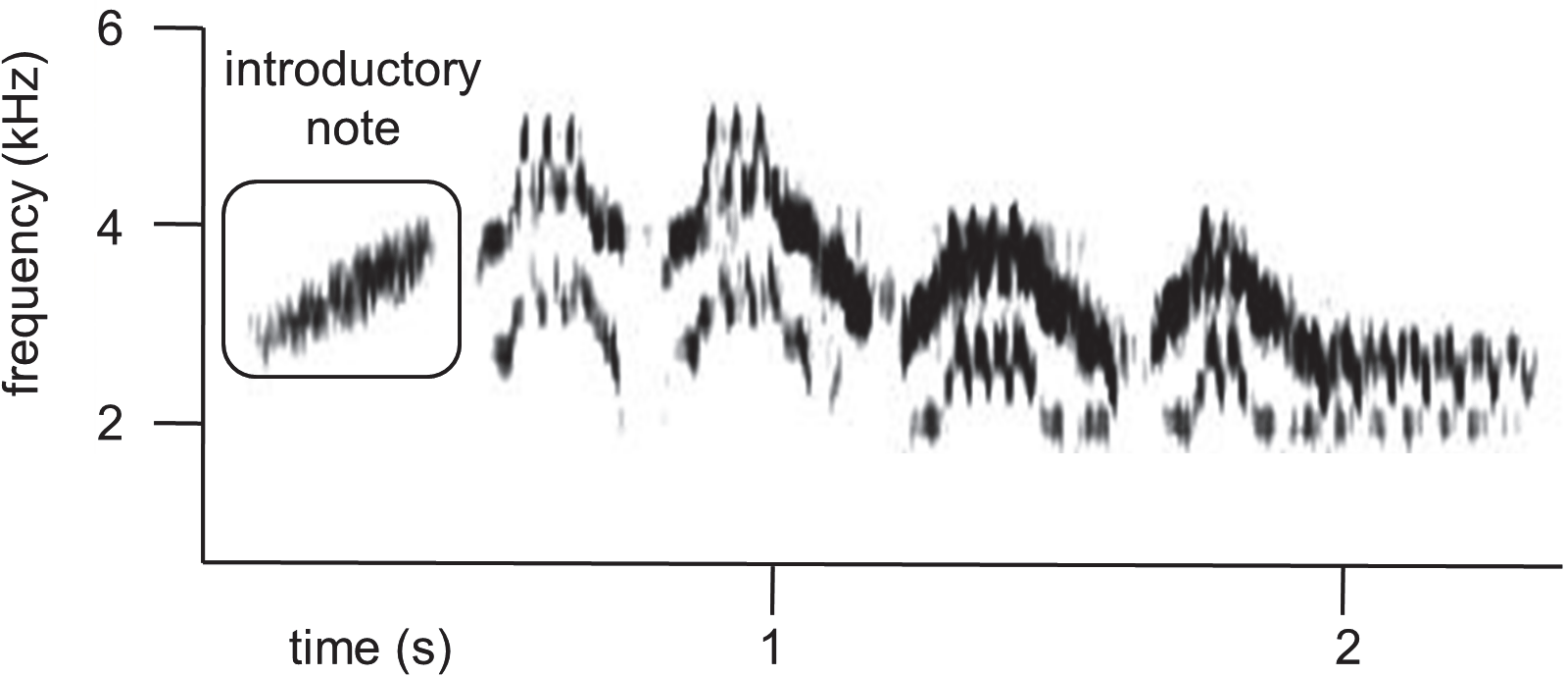
Sound spectrogram of a representative veery song. The rectangle identifies the introductory note, which veeries sometimes omit.

Veeries are reported to have small repertoires of one to two song types, although the songs are unusually complex in acoustic structure, and often feature multiple frequency bands sung simultaneously (Fig. 1). Veery song types vary enough to enable identification of individuals by their songs [27, 28], and songs can be divided into three components or phrases [28, 29]. The first phrase consists of one short ascending introductory note (highlighted in Fig. 1), the second phrase consists of a group of repeated notes that usually contain multiple frequency bands and often include the highest frequencies of the song, while the third phrase includes a second group of complex repeated notes shifted down in frequency [29]. Veeries also have a large repertoire of at least seven different calls, the functions of which are unclear [28].

**Fig 2:**
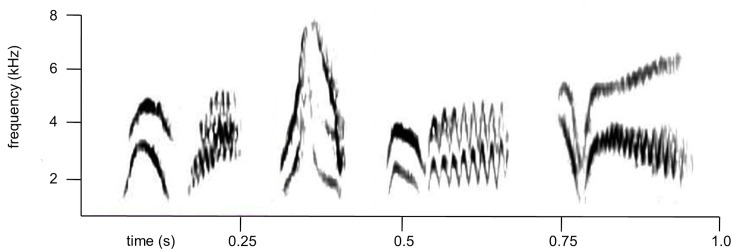
Sound spectrograms of four whisper calls (a-d) recorded from a single veery. Note the short length and large frequency range of this call, which we depict here for the first time.

Veery singing behavior includes several potential agonistic signals. Males frequently modify their songs by repeating and omitting notes from their usual song types during natural singing [28, 30]. We have observed that one type of modification, the singing of songs lacking the introductory note, seems to be more common in agonistic contexts, and thus we hypothesize that this type of song modification might be an agonistic signal in veeries. Dilger [31] observed the use of an unnamed vocalization described as a “faint, high-pitched, windy, squealing sound” in highly agonistic situations. We have also observed the use of an acoustically complex, low-amplitude vocalization (likely the same one Dilger described), which we refer to as the whisper call (Fig. 2). We refer to this vocalization as the whisper call because of its low amplitude and short duration. We measured the acoustic properties of 211 veery whisper calls, songs, and other veery calls (primarily “veer” calls) taken from natural recordings of eight focal males who remained relatively stationary while they were vocalizing and gave a mix of whisper calls, songs, and other calls. Amplitude (loudness) varies with distance, so measuring the amplitude of songs and calls produced while birds were stationary allowed us to compare relative differences in amplitude among the different vocalization types. The number of vocalizations measured for each focal male ranged from 10–55, with at least 1 whisper call and multiple songs measured for each male. A total of 57 whisper calls, 41 veer calls, and 113 songs were measured using visual selections for time and frequency measurements, and the automated maximum and average energy measurement tools for amplitude in Raven Pro 1.4 software for Mac. We found that whisper calls resemble songs in terms of their large frequency ranges, and that they resemble other calls in terms of their short lengths, but that they have significantly lower average (Wilcoxon normal approximation: *z* = −3.3 p = 0.0009) and maximum amplitudes (*z* = −3.3 p = 0.0009) than either songs or other calls (Table 1). Note that the absolute values for amplitude are not meaningful since they reflect the distance between the bird and the microphone, but that the relative differences in amplitude between whisper calls and the other vocalizations are informative. We have recorded whisper calls both in response to playback of recorded veery song and during natural counter-singing (S1 Audio). However, without experimental evidence, we cannot be certain whether or not songs lacking the introductory note or whisper calls are agonistic signals in this species.

**Table 1:** Comparison of the acoustic properties of three categories of veery vocalizations: Songs, non-whisper calls (primarily “veer” calls), and whisper calls.

Acoustic Property	Songs	Veer Calls	Whisper Calls
Low Frequency (Hz)	1457 ±86	1442 ±74	1480 ±97
High Frequency (Hz)	5459 ±100	3774 ±107	5546 ±206
Frequency Range (Hz)	4001 ±107	2332 ±169	4065 ±185
Length (s)	1.89 ±0.06	0.35 ±0.01	0.35 ±0.02
Mean Amplitude (db)	76 ±1.3	81 ±0.2	64 ±1
Maximum Amplitude (db)	101 ±1.2	99 ±0.2	83 ±1

All values represent means ± standard errors for mean values of vocalizations measured for each male.

Here we used two playback experiments to identify agonistic signals in the veery. In the first experiment, we identified those vocalizations that are used by the focal male in an aggressive context (playback of natural veery singing), and compared them to vocalizations used in response to two types of putative agonistic signals (playback of veery singing with whisper calls added and veery singing with some introductory notes removed). In the second experiment, we examined which focal vocalizations might predict subsequent attack of a taxidermic veery mount during a simulated escalating intrusion using multimodal agonistic signals (robotic mount and song playback with high levels of both putative agonistic signals). In response to the first experiment, we predicted that focal males would use whisper calls and songs lacking the introductory note more during playback of natural songs than before the playback began, and that males would respond differently to the different types of playback of natural songs, songs and whisper calls, and songs with some lacking the introductory note, possibly indicating varying levels of aggression. In response to the second experiment, we expected that males would again use whisper calls and songs lacking the introductory note more during playback of natural songs than before the playback began, and that males would either increase or decrease their use of these signals as the simulated intrusion intensified. We also predicted that males that used one or both of these signals the most or least would be most likely to attack the taxidermic mount.

## Methods

This study was conducted in an oak-maple dominated forests at the Cary Institute of Ecosystem Studies in Dutchess County, New York, U.S.A (41°50′ N, 73°45′ W). Permission to study veeries on the private grounds of the Institute was granted prior to each field season. For permission to access this site, researchers may contact the Manager of Field Research, Michael Fargione (fargionem@caryinstitute.org). Trials for the first experiment were conducted between 06:00 and 10:00, from May 24 to June 27, 2010. Trials for the second experiment were conducted two years later between 06:00 and 10:00, from May 15 to July 3, 2012. Subjects were male veeries from a population of breeding territorial birds that could be identified individually by either color bands and/or song types. All procedures were conducted according to a protocol approved by the Institutional Animal Care and Use Committee at Texas Tech University (protocol 08047-02).

### Playback stimuli

We constructed 32 playback stimuli from recorded samples of 32 different male veeries that we recorded previously. We constructed our stimuli from sequences of natural singing because little is known about the function of particular veery songs, although it is known that veeries frequently modify their songs from one rendition to the next [32]. Recordings were made on site either during May, June, or July 2009, or early in May 2010, using a Telinga parabolic reflector, a Sennheiser MKH 62 microphone and a Marantz PMD 660 digital recorder (sampling rate of 44.1kHz, bit rate of 705.5 kbps). We chose only high-quality (high signal to noise ratio) recordings exclusively made up of sequences exclusively containing complete songs (without calls) for use as the control stimuli. To avoid using a male’s own song as a stimulus, we did not used stimuli recorded in locations at least two or three territories away from the focal male (veeries at our site have high between-year site-fidelity).

Each stimulus set was composed of a one-minute song sample that was used as a control stimulus, and then three copies of each stimulus were made and modified to create the three treatment stimuli. Each one-minute control stimulus included similar numbers of songs (mean ± standard error: 9.7 ± 0.27 songs). We used Raven software (Cornell Laboratory of Ornithology, Ithaca, NY, USA) to filter out all noise surrounding each song and to standardize the average amplitudes of each stimulus (101.9 ± 0.095 db as measured by Raven’s “energy” measurement tool). To create the “whisper call” treatment stimuli for the first experiment, we added three different previously recorded whisper calls halfway between randomly chosen songs to mimic the way veeries mix whisper calls and songs in nature. Each whisper call was used only once and was selected from previously made recordings of natural singing and calling made in 2009 and in early May 2010. To create the “no introductory note” treatments for the first experiment, we removed the introductory note from three randomly selected songs using Raven’s “filter out” tool. To create the “high aggression” treatments for the second experiment, we added three more different previously recorded whisper calls halfway between randomly chosen songs to the files previously used as our “whisper call” treatments, and we removed the introductory note from six randomly selected songs in the same fashion as we did for the “no introductory note” treatment. This resulted in the creation of “high aggression” treatments containing twice the number of whisper calls and twice the number of songs without the introductory note than the stimuli we used in the first experiment. When each of these stimuli were played on the mp3 player through our playback speakers in the forest, we adjusted the volume of the speakers so that the stimuli played at ~85 db when measured at 1 m from the speaker using a sound level meter (Radio Shack).

### Playback trials

Before beginning trials in each experiment, we located an actively vocalizing male veery, and set up our equipment 30–40m away from the male. We tied a playback speaker and mp3 player one meter high in a tree and then sat 15m away from the experimental set-up for the duration of the trial. One observer (CEN in 2012 and several rotating assistants in 2010) used a parabolic recording set-up (Telinga parabolic reflector, a Sennheiser MKH 62 microphone and a Marantz PMD 660 digital recorder) to record all vocalizations from the focal male, while the other (KLB) followed the focal male’s movements with binoculars and narrated information about physical displays, color-bands, and distance from the speaker (aided by flags at 5 and 10m from the speaker).

**Fig 3:**
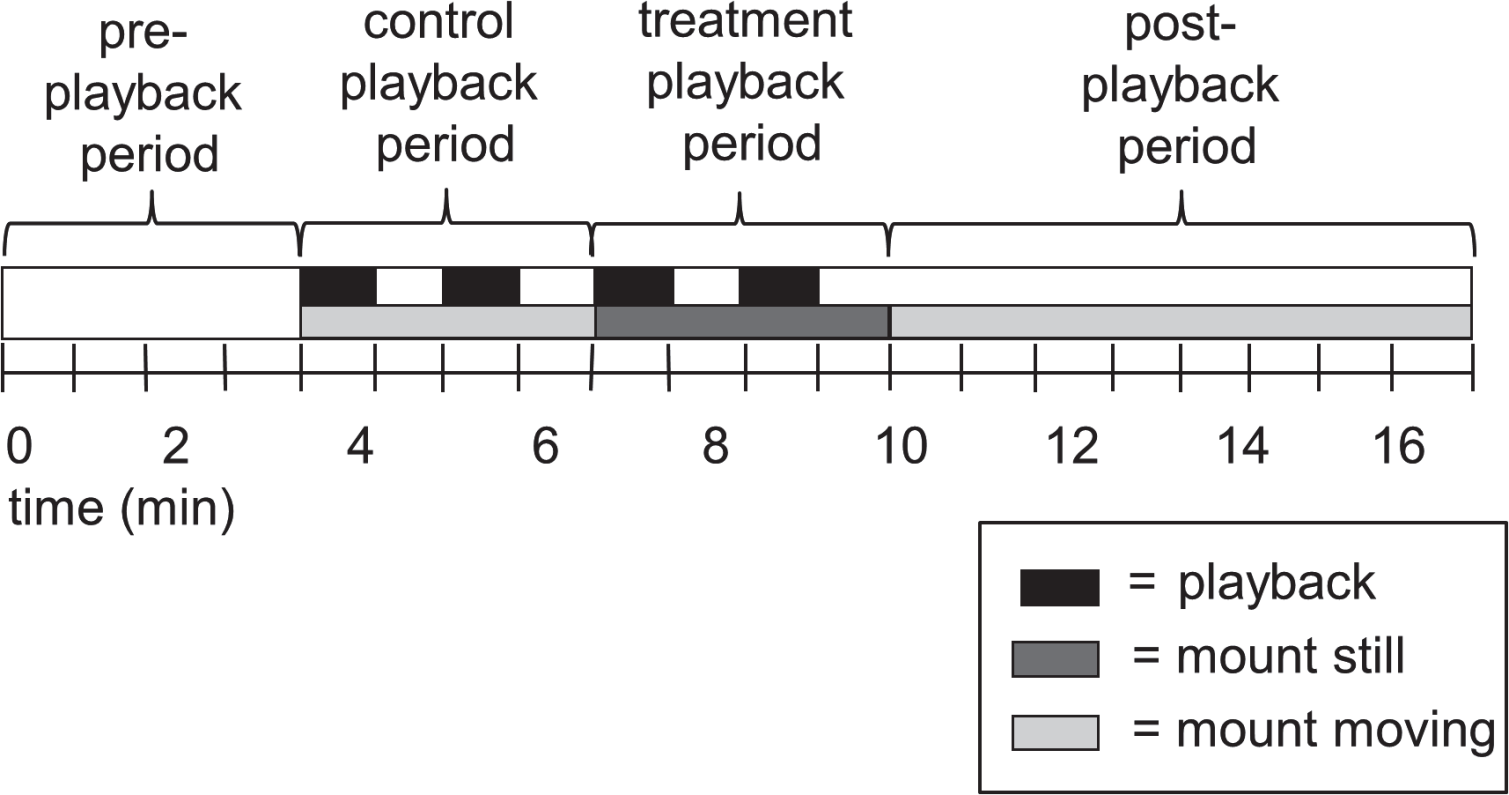
Schematic timeline of the playback experiment. The timing of acoustic playbacks (for both experiments), as well as the uncovering of the robotic mount and the timing of robotic movements (for the second experiment only) are indicated.

Each playback trial was 20 minutes long (Fig. 3). We began the trial by recording the focal male’s natural behavior during four minutes of silence (the pre-playback period), then transitioned to the control playback period, during which we played the one-minute control stimulus twice, leaving one minute of silence after each control stimulus. Next we entered the treatment period during which we played the one-minute treatment stimulus twice, leaving one minute of silence after each treatment stimulus. We finished the trial by recording the focal male’s behavior during eight more minutes of silence.

### Experiment #1 (acoustic playback treatment)

In the first experiment, each trial included a control stimulus composed of a sequence of natural veery songs and one of the two treatment stimuli: the “whisper call” treatment or the “no introductory note” treatment. We alternated beginning with either the whisper call or no introductory note treatment. If a trial was successful, we then repeated the trial the next day with the remaining treatment. On the second day, we set up the experiment in the same location as on the first day, but we did not wait for the focal male to vocalize. If we did not see or hear the focal male by the end of the control period, we repeated the control stimulus up to three times. If the male was seen or heard, we then completed the trial. If the male was not seen or heard, we abandoned the trial and returned to complete it the following day. Almost all trials were completed on consecutive days, although some were completed later if the male did not respond or the weather prevented the trial from being completed (mean ± standard error: 1.24 ± 0.08 days between trials).

### Experiment #2 (multimodal high-aggression treatment)

Playback trials in the second experiment were identical to those conducted in the first experiment, except that only a single trial was done with each focal male, and it consisted of the control playback and one multimodal treatment playback using an acoustic stimulus and the robotic mount (Fig. 3). The acoustic stimulus used for the treatment playback was the “high aggression” stimulus described previously. The robotic mount was made from a taxidermy skin of a male veery fitted onto a moveable body and with moveable plates under the wings attached to servo motors concealed in a box under the mount that also housed a transmitter to a radio-control remote (S1 Video). The robot was placed on a tripod approximately 1.5m off the ground and as close as possible to the speaker in order to create a more realistic stimulus and allow territorial birds a target on which to focus their attention. The robot is able to flick its wings and bow up and down, movements that approximate aggressive movements in *Catharus* thrush species, including veeries (Dilger 1956). In the field, the robotic mount was concealed with a camouflaged cloth attached to a string. At the end of the pre-playback period, we pulled the string to uncover the mount, but left it motionless as the control stimulus played. When the high aggression treatment was played, the observer who was narrating used the remote control to create a series of naturalistic movements, alternating between bows and wing-flicks in time with the playback songs and calls, to mimic the way we have observed veeries using these movements naturally.

### Analysis

We extracted data from the recordings of each trial by listening to our narration and scrolling through sound spectrographs produced in Raven. The song parameters we measured were number of full songs sung, the number of calls uttered (excluding whisper calls), the number of whisper calls uttered (See Fig. 2), and the number of songs sung lacking the introductory note (see Fig. 1). We used our narration of the movements of focal birds to count the number of times the focal male made quick flights low over the speaker (hereafter referred to as “swooping”). In both experiments, most of the behavioral data were not normally distributed (left-skewed due to many low and zero values), so we used nonparametric tests. We used Holmes’s sequential Bonferroni method for adjusting significance levels to correct for making multiple comparisons (correlations) with the same data set. All statistical analyses were performed using JMP 10.0.2 software (SAS Institute).

## Results

### Experiment #1 (acoustic playback treatment)

We initiated 32 trials, 2 of which we excluded from analysis due to interference from neighboring males. In the 30 trials we analyzed, all focal males sang and called at some point during the experiment (mean ± standard error: 22.5 ± 3.1 full songs, 9.2 ± 1.9 non-whisper calls). Males sang songs lacking the introductory note at least once in 20 of 30 trials and uttered whisper calls in 17 of 30 trials, although not all trials containing one vocalization also contained the other. Half of the focal males made one or more swoops over the speaker during the playback period. All but five males approached within 10m of the speaker during the playback period. Males frequently performed agonistic behaviors such as wing flicks and bill wiping, as well as behaviors indicating alert attention such as visual scanning (looking around for a rival) and flying quickly from perch to perch.

### Vocal behaviors used in aggressive contexts

**Fig 4:**
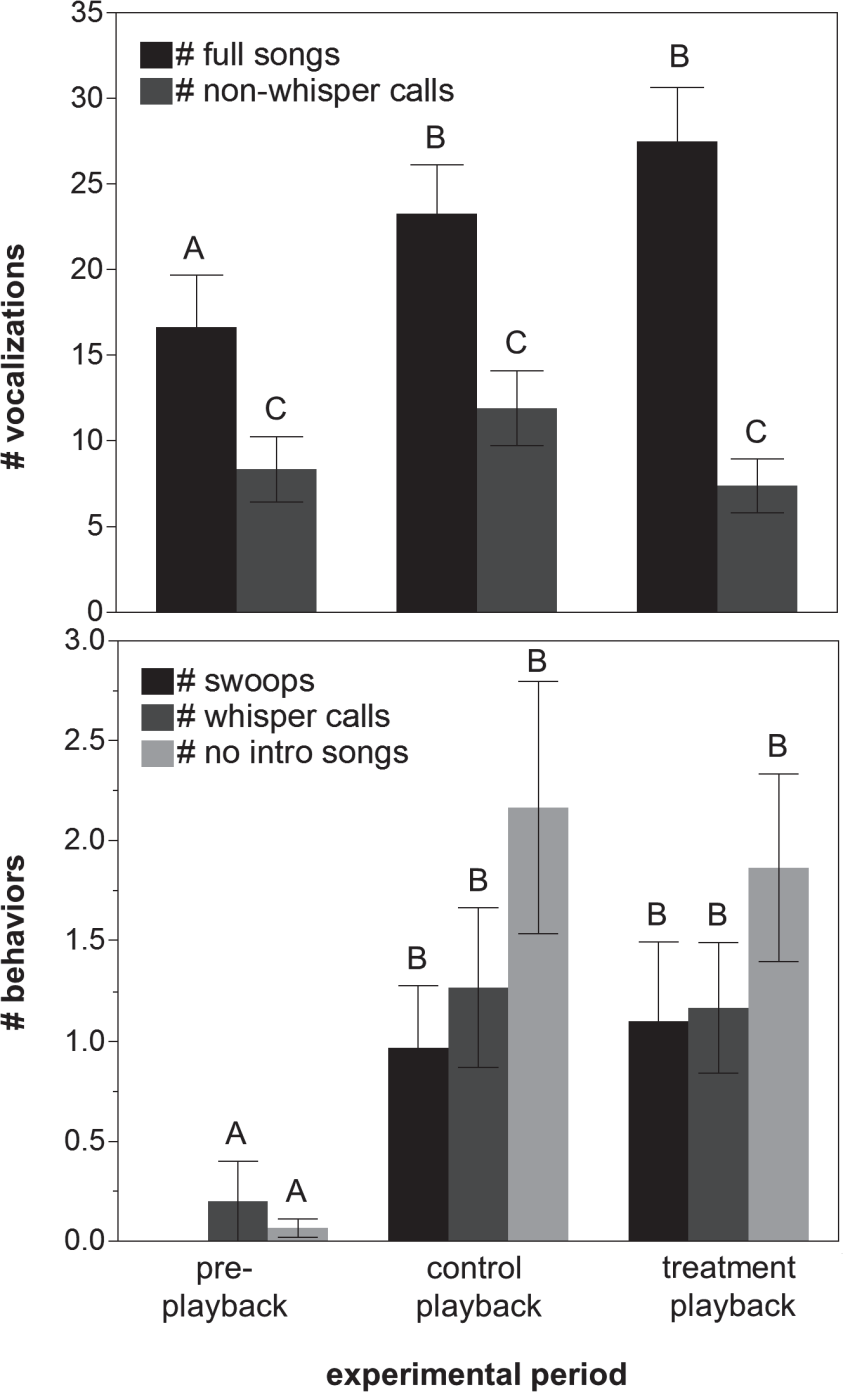
Veery behaviors observed during the pre-playback, control playback, and treatment playback periods. Behaviors include total songs and non-whisper calls (top panel), and swoops over the speaker, whisper calls, and songs without introductory notes (bottom panel). Different letters above each bar represent statistically different groups.

All males vocalized during set-up on the first trial date, although 8 of 30 males subsequently stopped vocalizing during the pre-playback period of the first experimental trial before vocalizing again during one or more of the playback periods. Nearly an equal number of males (9 of the 30) were silent during the pre-playback period of the second experimental trial before, again, vocalizing during one or more of the playback periods, indicating that beginning trials on the second day without waiting for the focal male to vocalize had little effect on male’s behavior during the pre-playback period. We took advantage of our paired experimental design by including focal male identity as a blocking factor in all of our analyses to correct for differences in vocalization rates among males. Across both trial dates, males uttered significantly different numbers of full songs, whisper calls, and songs without introductory notes across the three different periods of the experiment (Wilcoxon rank sum using Chi-square approximation: all p < 0.001). Males uttered marginally different numbers of non-whisper calls during the different playback periods (χ^2^_2_ = 5.76 p = 0.06, Fig. 4 top panel). More specifically, males sang more full songs during the control and treatment playback periods than they did during the pre-playback period (Wilcoxon normal approximations: pre-playback vs. control playback: *Z* = 3.13, N = 30, p = 0.0018, pre-playback vs. treatment playback: *Z* = 4.21, N = 30, p < 0.0001, Fig. 4 top panel). Birds also uttered significantly more whisper calls during the control and treatment playback period than they did during the pre-playback period (pre-playback vs. control playback: *Z* = 3.16, N = 30, p = 0.002, pre-playback vs. treatment playback: *Z* = 3.47, N = 30, p = 0.0005, Fig. 4 bottom panel). Finally, males sang significantly more songs lacking the introductory note during the control and treatment playback period than they did during the pre-playback period (pre-playback vs. control playback: *Z* = 4.26, N = 30, p < 0.0001, pre-playback vs. treatment playback: *Z* = 4.18, N = 30, p < 0.0001, Fig. 4 bottom panel).

### Vocal behaviors used in response to agonistic signals

**Fig 5:**
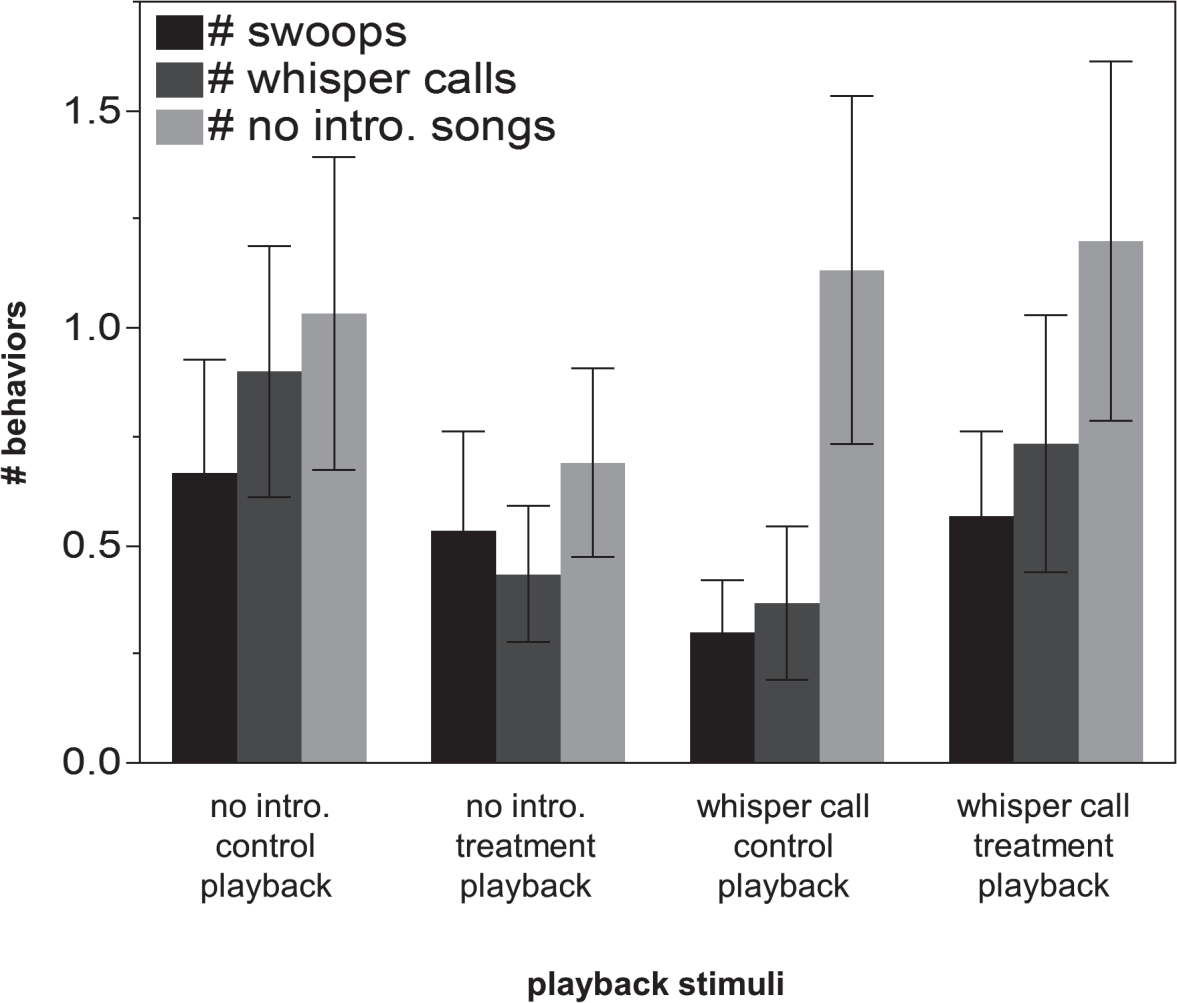
Veery behaviors observed during the different playback periods. The control playback period consisted of playback of naturally recorded songs, and the two treatment playback periods that included either recordings of songs with introductory notes removed or songs with whisper calls added. There were no significant differences in any of the behaviors among the different playback periods.

All males were spotted visually or produced vocalizations during the control and treatment playback periods in each trial. However, there were marginally significant differences in the number of full songs, and no significant differences in the number of whisper calls or songs without introductory notes given by focal males during the control playback period as compared to during the treatment playback period of the experiment (Wilcoxon normal approximations: *Z* = 1.92, N = 30, p = 0.055, Z = 0.131, N = 30, p = 0.8956, Z = 0.038, N = 30, p = 0.9697, full songs, whisper calls, and no introductory note songs respectively, Fig. 4). In addition, there were no differences in the number of full songs, whisper calls, or songs without introductory notes that males made among the two different control and two different (whisper calls added or introductory notes removed stimuli) treatment playback periods (χ^2^_2_ = 3.74 p = 0.2910, χ^2^_2_ = 4.92 p = 0.1781, χ^2^_2_ = 0.13 p = 0.9885, respectively, Fig. 5). There was a significant difference in the number of non-whisper calls produced in the different playback periods (χ^2^_2_ = 11.67 p = 0.009, Fig. 5); males produced significantly more non-whisper calls during the control playback period of the no introductory note trial than they did during the no introductory note treatment playback period of that trial (Wilcoxon normal approximations: *Z* = −2.63, N = 30, p = 0.009, Fig. 5).

### Vocal behaviors associated with physical aggression

**Fig 6:**
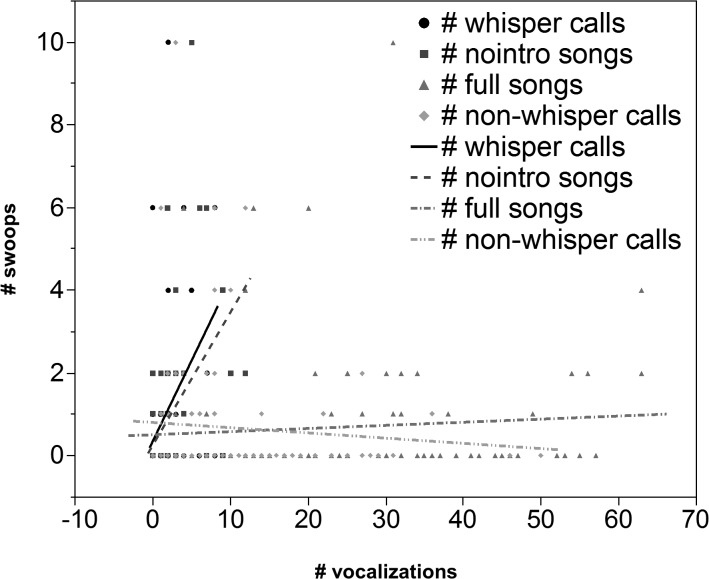
Veery vocal behaviors associated with aggressive swooping behavior. For vocalizations across all experimental periods, the number of swoops over the speaker is positively correlated with number of whisper calls (Spearman’s rank correlation: *p* = 0.5128, p < 0.0001) and the number of songs without the introductory note (*p* = 0.5805, p < 0.0001), but is not significantly related to the number of full songs or non-whisper calls given (p > 0.01, which is the significance cut-off for five comparisons). The number of whisper calls given is also highly correlated with the number of of songs without the introductory note (*p* = 0.6256, p < 0.0001).

Males behaved aggressively in many of our trials, and the most aggressive behavior we observed was a fast, low flight over the speaker, or a “swoop”. Half of all of our focal males swooped in at least one of their two trials, and males swooped average of 0.69 ± 0.30 times overall, with several males swooping 6–10 times. No males swooped during the pre-playback period, and so there was a highly significant difference between the number of swoops performed by males in the pre-playback period as compared to the control playback and treatment playback periods (pre-playback vs. control playback: *Z* = 3.39, N = 30, p = 0.007, pre-playback vs. treatment playback: *Z* = 3.73, N = 30, p = 0.0002, Fig. 4 bottom panel). Nearly equal numbers of males swooped during the control and treatment playback periods (11 and 12 respectively), and there were no differences in the number of swoops that males made among the two different control and treatment playback periods (χ^2^_2_ = 1.396 p = 0.715, Fig. 5). Across all playback periods, the number of swoops performed by a male was highly correlated with both the number of whisper calls, and the number of songs without introductory notes given by that male, and the numbers of these two vocalizations that were given were also highly correlated (Fig. 6). However, the number of swoops performed was unrelated to the number of full songs or non-whisper calls given overall (Fig. 6).

### Experiment #2 (multimodal high-aggression treatment)

We conducted 31 complete trials using the “high aggression” playback and the robotic mount, but we excluded three trials from analysis due to interference from neighboring males and one trial due to poor recording quality from stream noise. The number of full songs and non-whisper calls given by males in this experiment did not differ between the pre-playback, control playback and treatment playback periods (Wilcoxon rank sum using Chi-square approximation: both p > 0.1). Focal males responded to the multimodal stimuli by vocalizing similarly to the way they did in the first experiment in that they gave different numbers of whisper calls and songs without introductory notes during the different experimental periods (Wilcoxon rank sum using Chi-square approximation: all p < 0.001). Males produced more whisper calls (pre-playback vs. control playback: *Z* = 3.20, N = 27, p = 0.001, pre-playback vs. treatment playback: *Z* = 3.16, N = 27, p = 0.002) and no introductory-note songs (pre-playback vs. control playback: *Z* = 3.52, N = 27, p = 0.0004, pre-playback vs. treatment playback: *Z* = 3.34, N = 27, p = 0.0008) during the control and treatment playback periods as compared to during the pre-playback period. The numbers of whisper calls and songs without introductory notes given by males were again highly correlated (Spearman’s rank correlation: *p* = 0.5242, p < 0.0001). In addition, there were again no discernable differences in singing behavior between the control playback and “high-aggression” treatment playback periods (whisper calls control playback vs. treatment playback: *Z* = −0.02, N = 27, p = 0.9850, no intro. songs pre-playback vs. treatment playback: *Z* = −0.22, N = 30, p = 0.8287).

### Vocal Behaviors Predicting Aggression

No veeries attacked the robotic mount in any of our trials, although focal males were clearly agitated, and we observed many wings flicks, bill wipes, and quick flights from perch to perch. Unexpectedly, far fewer males swooped over the speaker and the mount during this experiment than they had during the first experiment. Only two males swooped during this experiment (one of these males swooped once and the other swooped twice). Four males failed to approach closer than 10m from the speaker and the mount. One male did not sing once the trial began, and two males went silent after the pre-playback period and were not seen again during the experiment. Many of the males who remained made short flights from perch to perch in the vicinity (within 10m) of the speaker and mount. In an effort to lure at least one focal male into attacking the mount, we repeated our high aggression stimuli with robotic movements for various lengths of time after some of the trials during which the focal male had appeared to be fairly aggressive, but we were never able to incite any kind of physical contact between a focal male and the mount.

## Discussion

In our first experiment, we found that veeries readily responded to our simulation of a territorial intrusion. During playback, veeries sang at an increased rate, and many birds swooped over the speaker, indicating that the veeries perceived the experiments as agonistic situations. In addition, we found that veeries used whisper calls and songs without introductory notes in aggressive contexts more than during undirected singing. Moreover, the use of whisper calls and songs without introductory notes were highly correlated with each other and with physically aggressive swooping behavior, which was only observed in response to playback. Taken together, we find these results to be convincing evidence that whisper calls and songs without introductory notes are agonistic signals in this species. However, numbers of these behaviors observed did not differ between the control playback periods and any of our treatment playback periods that included whisper calls and songs without introductory notes, so we are unable to identify the receiver’s response to these signals. Therefore, these signals do not fulfill the “response criterion” defining signals of aggressive intent [5]. At the end of this first experiment, we concluded that since the number of whisper notes or introductory notes removed in our treatment stimuli were low compared to the numbers we observed many focal males producing during the experiment, our treatment stimuli were not sufficiently aggressive to elicit a response that differed from the response we observed during the control stimuli of natural songs. We designed the “high aggression” stimuli for our second experiment to mimic rates of whisper calls and songs without introductory notes that we observed in this experiment.

In our second experiment, we confirmed that veeries use whisper calls and songs without introductory notes in an aggressive context, and that these two signals were correlated with each other. However, we again found no difference in vocal behaviors given in response to the control stimuli as compared with our “high aggression” stimuli, and no veeries physically attacked the robotic mount. These results again indicate that whisper calls and songs without introductory notes fulfill the “context criterion” for aggressive signals, but again do not fulfill the “response criterion,” and we were unable to test whether or not they fulfill the “predictive criterion” since the birds did not attack [5]. We intended for the control stimuli to represent the beginning of a stimulated agonistic interaction and the treatment stimuli to represent an escalation of this interaction. However, based on the results form both experiments, it is possible that each male began signaling his level of aggressive motivation or ability very quickly during the control stimuli and then simply maintained this level of signaling. It is also possible that our treatment stimuli were lacking some important but subtle signals of escalation such as appropriate syntax or timing. Alternatively, it is possible that whisper calls and songs lacking the introductory note are agonistic signals of submissive intent, appeasement, or de-escalation, although this seems unlikely because they are correlated with seemingly aggressive swooping behavior.

Without an act of physical aggression, it is difficult to distinguish between signals of aggressive intent and other signals that may be used during agonistic interactions, such as signals of submission or de-escalation. Although it is possible that our robotic mount was not threatening or realistic enough to provoke attack, we find this to be a surprising explanation because Dilger [31] reported eliciting aggression from veeries using a paper mâché model, although he does not specify what kinds of playback stimuli he used, nor the details of the aggression displayed by the focal birds. Perhaps our multimodal stimuli were too threatening for the focal males to risk many swoops or a physical attack, or perhaps veeries simply resort to physical aggression less often than other songbird species known to attack models, such as sparrows, warblers, chickadees, and wrens [19, 21–23, 33–35]. Indeed we have rarely observed natural physical aggression among veeries, although intense counter-singing is common, and we have observed perch displacements and chases on occasion. As more species are tested for signals of aggression using taxidermic mounts, it is important to note that this method may simply not work for all species.

In this study, we experimentally identify a call, the whisper call, as an agonistic signal. Ballentine et al. [19] and Hof and Hazlett [21] tested for the possibility of several calls functioning as aggressive signals in swamp sparrows, *Melospiza georgiana*, and black-throated blue warblers, *Dendroica caerulescens*, but none of the calls observed in these studies were related to aggression. Baker et al. [33] identified the non-song gargle call as an aggressive signal predicting attack of a taxidermic mount in black-capped chickadees. The structure of these gargle calls is similar in acoustic complexity to the veery whisper call, although gargle calls are sung at similar amplitudes to broadcast songs, whereas veery whisper calls are sung at lower amplitudes. Veery whisper calls often contain simultaneous dual frequencies and many quick frequency modulations (Fig. 2). Individual male veeries sing several different whisper calls, primarily uttering these calls between songs, and occasionally replacing the introductory note of their song with a whisper call. Whisper calls are different from other veery calls in that they are given at low amplitudes, which may be why they have not been described in previous studies (but see [31]). Due to their low amplitudes, whisper calls may be an interesting parallel to aggressive soft song observed in sparrows and new and old-world warblers. Researchers have hypothesized that soft song may be an honest signal of aggressive motivation (escalation) for a variety of reasons including that low-amplitude vocalizations prevent a sender from attracting mates or defending against distant rivals, that they allow the sender to avoid eavesdropping by distant rivals and predators, that they can only be used in close proximity to a rival, which leaves the sender vulnerable to attack or makes retaliation more likely, or that males are physiologically unable to produce full song immediately before an attack [36, 37].

Early observational and experimental studies of veery song describe veeries singing songs lacking the introductory note, and songs with additions or omissions of other notes, as well [32, 38, 39]. Here, we find that the production of songs lacking the introductory note is used as an agonistic signal. The production of songs lacking the introductory note may be an example of a conventional signal of fighting motivation or ability, which may amplify or emphasize the use of the whisper call, although the mechanism for enforcing the honesty of this signal is unclear. Alternatively, songs lacking introductory notes might modify the signal of the whisper call in some other way; for example, songs without introductory notes may temper the aggressiveness of the whisper call. In our experiment, veeries did not respond differently to playbacks of songs with whisper calls added or playback of songs with introductory notes removed than they did to playback of natural songs, but we did not compare the effect of different mixtures of whisper calls and songs without introductory notes to test for interactions between the functions of these two signals. Future studies could include more refined experiments of this type to further delineate the functions of the two novel agonistic signals identified here in the veery: whisper calls and songs without introductory notes.

## Supporting Information

S1 Table Raw data from Experiment #1, Acoustic Playback Treatments. The table summarizes the number of each type of behavioral response for each of the 30 focal male during the different periods of the playback experiment. The mean and Standard Error for each behavior are provided ta the bottom of the table.
(DOCX)

S2 Table Raw data from Experiment #2, Multimodal High-aggression Treatments. The table summarizes the number of each type of behavioral response for each of the 27 focal males during the different periods of the playback experiment. The mean and Standard Error for each behavior are provided ta the bottom of the table.
(DOCX)

S1 Audio Natural Recording of a Male Veery Producing Songs and Whisper Calls. (WAV)

S1 Video Veery Robotic Taxidermic Mount. (MOV)
