# Supplementary material for: Two Novel Vocalizations Are Used by Veeries (Catharus fuscescens) during Agonistic Interactions
Source: PLoS One. 2015 Mar 23;10(3):e0120933. doi: 10.1371/journal.pone.0120933 (PMC4370839; doi:10.1371/journal.pone.0120933)
Supplement: S2 Table — The table summarizes the number of each type of behavioral response for each of the 27 focal males during the different periods of the playback experiment. The mean and Standard Error for each behavior are provided ta the bottom of the table. (DOCX) [file pone.0120933.s002.docx]

S2 Table. Raw data from Experiment #2, Multimodal High-aggression Treatments. The table summarizes the number of each type of behavioral response for each of the 27 focal males during the different periods of the playback experiment. The mean and Standard Error for each behavior are provided ta the bottom of the table.

| **trial number/focal male** | **experimental period** | **# full songs** | **# non-whisper calls** | **# nointro. songs** | **# whisper calls** | **# swoops** |
| --- | --- | --- | --- | --- | --- | --- |
| 1 | pre-playback | 2 | 0 | 0 | 0 | 0 |
| 2 | pre-playback | 15 | 3 | 0 | 0 | 0 |
| 3 | pre-playback | 23 | 11 | 0 | 0 | 0 |
| 4 | pre-playback | 33 | 1 | 0 | 0 | 0 |
| 5 | pre-playback | 43 | 0 | 0 | 0 | 0 |
| 6 | pre-playback | 0 | 0 | 0 | 0 | 0 |
| 7 | pre-playback | 5 | 31 | 0 | 0 | 0 |
| 8 | pre-playback | 0 | 0 | 0 | 0 | 0 |
| 9 | pre-playback | 5 | 26 | 0 | 0 | 0 |
| 10 | pre-playback | 28 | 20 | 0 | 0 | 0 |
| 11 | pre-playback | 0 | 46 | 0 | 0 | 0 |
| 12 | pre-playback | 0 | 0 | 0 | 0 | 0 |
| 13 | pre-playback | 10 | 7 | 0 | 0 | 0 |
| 14 | pre-playback | 0 | 42 | 0 | 0 | 0 |
| 15 | pre-playback | 28 | 17 | 0 | 2 | 0 |
| 16 | pre-playback | 20 | 7 | 0 | 0 | 0 |
| 17 | pre-playback | 6 | 43 | 0 | 1 | 0 |
| 18 | pre-playback | 0 | 12 | 0 | 0 | 0 |
| 19 | pre-playback | 24 | 15 | 0 | 0 | 0 |
| 20 | pre-playback | 0 | 22 | 0 | 0 | 0 |
| 21 | pre-playback | 7 | 14 | 0 | 0 | 0 |
| 22 | pre-playback | 8 | 44 | 0 | 0 | 0 |
| 23 | pre-playback | 0 | 29 | 0 | 0 | 0 |
| 24 | pre-playback | 0 | 0 | 0 | 0 | 0 |
| 25 | pre-playback | 14 | 9 | 0 | 0 | 0 |
| 26 | pre-playback | 0 | 20 | 0 | 0 | 0 |
| 27 | pre-playback | 11 | 25 | 1 | 3 | 0 |
| 1 | control playback | 0 | 0 | 0 | 0 | 0 |
| 2 | control playback | 11 | 4 | 0 | 0 | 0 |
| 3 | control playback | 3 | 0 | 13 | 0 | 0 |
| 4 | control playback | 4 | 18 | 3 | 1 | 0 |
| 5 | control playback | 27 | 2 | 2 | 1 | 0 |
| 6 | control playback | 1 | 2 | 0 | 0 | 0 |
| 7 | control playback | 28 | 17 | 1 | 0 | 0 |
| 8 | control playback | 0 | 0 | 0 | 0 | 0 |
| 9 | control playback | 18 | 1 | 1 | 0 | 0 |
| 10 | control playback | 37 | 0 | 0 | 0 | 0 |
| 11 | control playback | 14 | 36 | 3 | 15 | 0 |
| 12 | control playback | 9 | 0 | 3 | 0 | 0 |
| 13 | control playback | 19 | 10 | 3 | 2 | 0 |
| 14 | control playback | 23 | 49 | 0 | 1 | 0 |
| 15 | control playback | 9 | 5 | 0 | 0 | 0 |
| 16 | control playback | 0 | 0 | 0 | 0 | 0 |
| 17 | control playback | 9 | 18 | 2 | 3 | 0 |
| 18 | control playback | 27 | 1 | 1 | 1 | 1 |
| 19 | control playback | 16 | 15 | 2 | 2 | 0 |
| 20 | control playback | 15 | 7 | 0 | 2 | 0 |
| 21 | control playback | 0 | 51 | 0 | 0 | 0 |
| 22 | control playback | 1 | 8 | 10 | 3 | 0 |
| 23 | control playback | 1 | 18 | 0 | 0 | 0 |
| 24 | control playback | 30 | 0 | 0 | 0 | 0 |
| 25 | control playback | 1 | 4 | 0 | 0 | 0 |
| 26 | control playback | 16 | 24 | 0 | 0 | 0 |
| 27 | control playback | 12 | 30 | 2 | 6 | 0 |
| 1 | treatment playback | 12 | 1 | 1 | 0 | 0 |
| 2 | treatment playback | 13 | 2 | 0 | 0 | 0 |
| 3 | treatment playback | 0 | 0 | 11 | 0 | 0 |
| 4 | treatment playback | 6 | 2 | 11 | 1 | 0 |
| 5 | treatment playback | 35 | 2 | 0 | 0 | 0 |
| 6 | treatment playback | 0 | 0 | 0 | 0 | 0 |
| 7 | treatment playback | 20 | 7 | 3 | 1 | 0 |
| 8 | treatment playback | 0 | 2 | 0 | 0 | 0 |
| 9 | treatment playback | 28 | 2 | 0 | 0 | 0 |
| 10 | treatment playback | 35 | 0 | 0 | 0 | 0 |
| 11 | treatment playback | 21 | 21 | 1 | 10 | 1 |
| 12 | treatment playback | 21 | 0 | 1 | 0 | 0 |
| 13 | treatment playback | 4 | 4 | 0 | 0 | 0 |
| 14 | treatment playback | 8 | 5 | 4 | 2 | 0 |
| 15 | treatment playback | 5 | 1 | 0 | 0 | 0 |
| 16 | treatment playback | 0 | 24 | 0 | 0 | 0 |
| 17 | treatment playback | 2 | 14 | 5 | 12 | 0 |
| 18 | treatment playback | 14 | 2 | 2 | 2 | 1 |
| 19 | treatment playback | 7 | 18 | 0 | 0 | 0 |
| 20 | treatment playback | 19 | 1 | 4 | 1 | 0 |
| 21 | treatment playback | 5 | 12 | 0 | 0 | 0 |
| 22 | treatment playback | 6 | 4 | 3 | 1 | 0 |
| 23 | treatment playback | 0 | 3 | 0 | 2 | 0 |
| 24 | treatment playback | 11 | 36 | 0 | 0 | 0 |
| 25 | treatment playback | 3 | 61 | 0 | 0 | 0 |
| 26 | treatment playback | 15 | 38 | 0 | 0 | 0 |
| 27 | treatment playback | 5 | 39 | 1 | 9 | 0 |
|  | **mean** | **11.2** | **13.1** | **1.2** | **1.0** | **0.0** |
|  | **SE** | **2.0** | **2.8** | **0.5** | **0.5** | **0.0** |
